# Temporal Trends in Medical and Surgical Management of Ulcerative Colitis in England: 2003–2020

**DOI:** 10.1111/apt.70319

**Published:** 2025-08-08

**Authors:** J. Couch, K. Thomas, T. R. Card, D. J. Humes

**Affiliations:** ^1^ National Institute for Health Research Nottingham Digestive Diseases Biomedical Research Centre Nottingham University Hospitals and University of Nottingham Nottingham UK

**Keywords:** colectomy, biological products, healthcare trends, ulcerative colitis

## Abstract

**Background:**

The medical management of ulcerative colitis (UC) is evolving. However, colectomy may be required in severe or refractory cases.

**Aim:**

To provide contemporary evidence on medication usage and surgery for UC.

**Methods:**

An incident cohort of patients newly diagnosed with UC from 2003 to 2020 was identified using computerised health records. The cumulative incidence of colectomy and medication use was calculated using Kaplan–Meier methods and compared across 3 time periods. We calculated 90‐day post‐operative mortality using life tables. Cox regression was used to model the risks of surgery and mortality, adjusting for confounders.

**Results:**

33,464 subjects had an incident diagnosis of UC. 5‐ year cumulative incidence of elective colectomy reduced from 2.97% to 1.59%, and emergency colectomy from 3.90% to 2.67% from 2003–2007 to 2015–2020. For elective surgery, adjusted hazard ratio (aHR) 0.47 and for emergency surgery, aHR 0.60 when 2015–2020 was compared to 2003–2007. Colectomy was less likely in women (elective aHR 0.73, emergency aHR 0.71) and emergency colectomy was less likely in those aged 40–59, aHR 0.87 than in those aged 18–39. Ninety‐ day mortality for elective and emergency surgery was 1.47% and 3.81%, respectively. Use of advanced therapies increased from 2.53% to 16.71% at 5 years from diagnosis when comparing 2003–2007 and 2015–2020.

**Conclusion:**

Colectomy in the five years following diagnosis has declined, coinciding with an increased use of advanced therapies. Overall post‐operative mortality is low. While the indication for colectomy may influence the risk of adverse outcomes, aggregate data provide a reassuring picture of current practice.

## Graphical abstract

Colectomy rates in ulcerative colitis have declined over the last two decades, coinciding with increased use of advanced therapies. These trends reflect significant changes in treatment approaches and provide valuable data to support informed decision‐making by patients, clinicians, and healthcare providers.

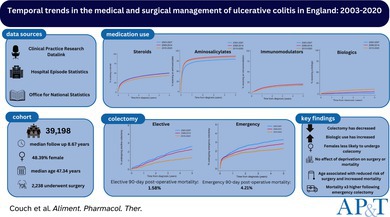

## 1 Introduction

Ulcerative colitis (UC) is a chronic disease with a variable disease presentation and clinical course that predominantly affects those of working age [1]. Medical and surgical treatments required to treat the disease have direct and indirect economic consequences and significantly impact quality of life [2, 3]. Patients with a new diagnosis of UC face uncertainty about how their treatment may escalate and the likelihood of needing medical or surgical intervention. The management of UC has changed considerably over the last 20 years with the widespread adoption of advanced therapies, advances in minimally invasive surgery and the implementation of IBD UK audit standards. These standards serve as benchmarks for healthcare providers, promoting safe, consistent and high‐quality care across the UK. For example, newly diagnosed patients with moderate disease are now expected to begin treatment within 48 h [4].

Colectomy rates reported from population‐based studies following a diagnosis of UC are variable [5, 6, 7, 8]. Even recently published systematic reviews do not report consistent findings [9, 10]. Despite being population‐based studies, the majority of studies utilise relatively small cohorts and do not differentiate between elective and emergency surgery. It is well established that planned and unplanned surgeries have different implications for patients in terms of morbidity and mortality, and for healthcare systems in terms of cost and resource allocation.

There is also conflicting evidence about the influence of advanced therapies on colectomy for UC [9, 11, 12, 13]. Data from the UK regarding the medication used for UC is limited despite concerns being raised about the appropriateness of steroid prescriptions issued in primary care [14] and escalating treatment costs in the post‐biologic era [15, 16]. Both surgery and medical therapies can have a profound effect on quality of life. Colectomy, though considered a definitive treatment, carries a risk of complications, particularly when performed in conjunction with an ileoanal pouch, and there is conflicting evidence as to whether surgery results in a long‐term improvement in quality of life for patients with UC [17]. Conversely, medication requires long‐term adherence, may have associated side effects, and has variable efficacy, which may ultimately result in surgery. In select cases, such as acute severe UC or refractory disease, where surgery is likely to be considered as an option, patients may find information regarding the likelihood of requiring surgery and its safety helpful when considering long‐term management options. Contemporary data regarding medication usage and the need for surgery to treat ulcerative colitis are necessary to guide clinicians and patients on the likely disease course and treatment progression following a new diagnosis of UC. It is also important for stakeholders involved in the development and delivery of IBD services to consider how the management of the disease is evolving so that resources can be acquired and allocated accordingly. The primary aim of this paper is to describe how colectomy rates and medication usage have changed over time. Secondary aims are to quantify mortality rates following surgery and determine risk factors for colectomy and post‐operative mortality.

## 2 Methods

### 2.1 Data Source

#### 2.1.1 Clinical Practice Research Datalink

Clinical Practice Research Datalink (CPRD) is a database that routinely collects data from subscribed general practices (GPs) in England. It includes anonymised patient‐level data on symptoms, investigations, prescriptions and diagnosis. As of March 2024, it encompassed over 16 million patients (24.15% of UK population) from 1784 GP's (19.86%) [18] and has been shown to be representative of the UK population when compared to census data [19]. Before being made available for research purposes, over 900 checks are made to ensure quality and validity of the data [20].

#### 2.1.2 Hospital Episode Statistics

Hospital Episode Statistics (HES) collects data from all NHS‐funded admissions in England, including those that take place in the private sector. It is estimated that 98%–99% of hospital care is financed by the NHS, making it largely representative of hospital care provided in England [21]. Data is collected on patient demographics, admission method, diagnoses, and procedures. Between June 2022 and May 2023, there were 16.4 million hospital admissions and over 11.8 million procedures recorded [22].

#### 2.1.3 Office for National Statistics

Office for National Statistics (ONS) mortality data provides essentially complete coverage of deaths in the UK, as death registration is a legal requirement. It also contains information on the place and cause of death as documented on the death certificate, an advantage over HES mortality data which only captures in‐hospital deaths and can be analysed by primary diagnoses rather than any underlying cause of death [23].

#### 2.1.4 Linked Data

Patient‐level data from CPRD can be linked to several healthcare‐related databases including HES and ONS to create a more comprehensive dataset. Data linkages are carried out by a third party, NHS digital, utilising deterministic linkage which uses rules based on agreement between identification variables [24].

### 2.2 Study Cohort

#### 2.2.1 Inclusion Criteria

An incident cohort was defined by identifying the first coded diagnosis in CPRD or HES of inflammatory bowel disease (Appendix S1 contains the list of codes utilised), a method which has previously been validated for research into IBD epidemiology [25]. We accepted as UC, cases with codes specifically for UC who also had additional generic IBD/indeterminate colitis codes. Cases with additional code(s) for Crohn's disease were accepted as UC only if their most recent recorded IBD code was specific for ulcerative colitis. The date of the first diagnostic code was considered to be the date of diagnosis. Diagnosis dates between 1st January 2003 and 31st December 2020 that were assigned to patients > 18 years of age were included.

#### 2.2.2 Exclusion Criteria

As historic diagnoses may not be immediately recorded on transfer to a new practice, we excluded as potentially prevalent cases with a first IBD diagnosis within 12 months of their practice registration start date [26]. Cases with a previous diagnosis of colorectal cancer or transplant, or a diagnosis based on a single IBD code recorded at the time of a day case endoscopy were also excluded.

### 2.3 Outcome Measures

Within HES data, colectomy was identified using Surveys Classification of Interventions and Procedures 4th revision (OPCS‐4) colectomy codes (Appendix S2) and the route of admission associated with the ‘spell’ number was also extracted. Colorectal cancer (CRC) as an indication for colectomy was defined as an incident ICD‐10 code for CRC occurring after the IBD diagnosis date and prior to the date of colectomy. Ninety‐day post‐operative mortality was assessed as all‐cause mortality and calculated from the date of surgery using the date of death recorded in ONS‐linked data. Medication use for oral steroids, aminosalicylates (oral and per rectal), immunomodulators (azathioprine, ciclosporin and tacrolimus) and advanced therapies was obtained from prescription records in CPRD using product codes. The use of biologics was acquired from HES using OPCS‐4 codes for therapeutic infusions, monoclonal antibodies, and cytokine inhibitors (Appendix S3). Prescriptions were counted if they occurred after the diagnosis date. For those who underwent surgery, prescriptions were not counted after the date of surgery.

### 2.4 Co‐Variates

Co‐variates were selected based on their established association with the likelihood of undergoing surgery and the risk of post‐operative mortality. They were included in our regression models to control for potential confounding. Gender was defined as male or female and regions by geographic areas in CPRD. Levels of deprivation were defined using the Index of Multiple Deprivation (IMD) which is a composite measure covering several domains of material deprivation. This study used small area level data, which is based on the postcode of the registered practice and categorised as levels of deprivation from 1 (least deprived) to 5 (most deprived). Age was grouped into 4 categories: 18–39, 40–59, 60–79, and greater than 80 years of age, based on age at the time of diagnosis or, in the case of mortality, the age at the time of surgery. Level of co‐morbidity was defined using the Charlson co‐morbidity index based on diagnostic codes recorded in HES and CPRD [27]. A composite score of 0, 1, or greater than or equal to 2 was assigned to each patient based on the level and severity of co‐morbidity. In order to compare time periods over the duration of the study, 3 time periods were defined: 2003–2007, 2008–2014, and 2015–2020. These were determined based on the National Institute for Health and Care Excellence (NICE) issuing guidance regarding the use of advanced therapies [28, 29] in order to elicit the effect of these medications on colectomy and the use of other medications. Medication use was defined if the patient had ever had a prescription for a steroid, aminosalicylate, immunomodulator, or advanced therapy following their diagnosis of UC. For 90‐day mortality, emergency or elective surgery was defined by the route of admission for the corresponding surgery assigned in the HES data.

### 2.5 Statistical Analysis

The demographics of the cohort were summarised using descriptive statistics. We used the Chi‐squared test to examine the variation of demographic variables between those who underwent colectomy and those who did not. The cumulative incidence of colectomy and first prescription were estimated using Kaplan–Meier methods. Surgical patients were censored at the date of surgery, date of death, or last recorded data collection at the registered practice and analysed by route of admission. For time to first prescription, study patients were censored at the date of first prescription, date of death, last recorded data collection at the registered practice, or date of surgery as colectomy is generally considered curative, and it would be standard UK practice to cease medical therapies [30]. Time to first surgery and prescription were examined in time periods based on the date of diagnosis (2003–2007, 2008–2014 and 2015–2020) which coincided with the National Institute for Health and Care Excellence (NICE) issuing guidance regarding the use of advanced therapies [28, 29]. Ninety‐day mortality was calculated using life tables. Cox proportional hazard models adjusted for the extracted covariates (see above) were produced to evaluate the association between co‐variables and the risk of elective colectomy, emergency colectomy, and 90‐day mortality.

Variables demonstrating statistical significance (*p* < 0.05) on univariate analysis were included in final models. Proportional hazards assumptions were tested using log–log survival plots and Schoenfeld residuals. All analyses were performed using Stata SE version 18 (64 Bit) software package (StataCorp. 2023. *Stata Statistical Software: Release 18*. College Station, TX: StataCorp LLC).

This study was approved by the Independent Scientific Advisory Committee of the UK Medicines and Healthcare products Regulatory Agency (MHRA) (protocol number 22_001723).

## 3 Results

### 3.1 Study Population

**FIGURE 1:**
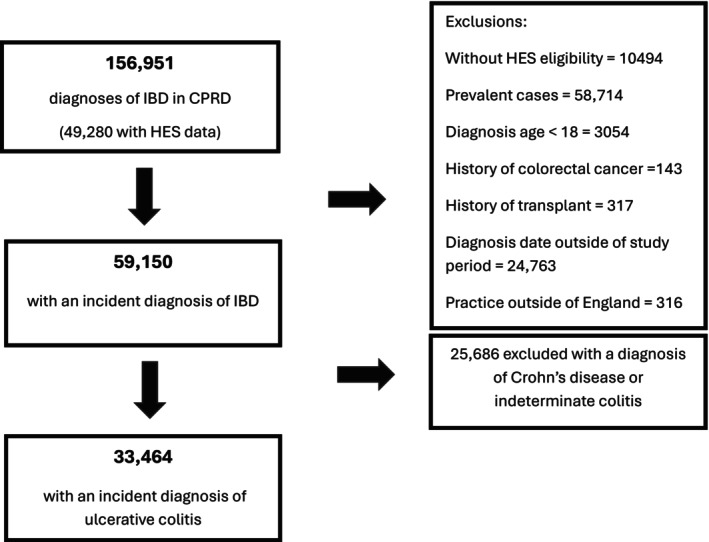
Study cohort flowchart. The flowchart outlines sequential steps outlining how the incident cohort was defined. There were 156,951 diagnoses of inflammatory bowel disease (IBD) identified from the Clinical Practice Research Datalink (CPRD) database. After applying exclusion criteria, there was 59,150 with an incident diagnosis of IBD, 33,464 were determined to be ulcerative colitis.

We identified 33,464 patients with an incident diagnosis of ulcerative colitis between 2003 and 2020 (Figure 1). 48.34% (16,176/33,464) were female. The median age of diagnosis for our adult cohort was 46.00 years (IQR 32.45–61.73 years). The median follow‐ up time was 8.27 years (IQR 4.56–13.17). 2236 underwent surgery, 54.96% (1229/2236) of which was via an emergency admission. Univariate Chi‐ squared analysis showed a significant association between sex, Charlson co‐ morbidity index, region, and age at diagnosis and likelihood of colectomy (Table 1).

**TABLE 1:** Demographics of cohort.

	Colectomy (*n* = 2236)	%	No colectomy (*n* = 31,238)	%	Total (*n* = 33,464)	%	χ^2^ (*p*)
**Gender**							
Male	1341	59.97	15,947	51.07	17,288	51.66	*p* < 0.0001
Female	895	40.03	15,281	48.93	16,176	48.34	
**Index of multiple deprivation level**							
1	377	16.86	5419	17.35	5796	17.32	*p* = 0.292
2	358	16.01	5446	17.44	5804	17.34	
3	473	21.15	6604	21.15	7077	21.15	
4	496	22.18	6763	21.66	7259	21.69	
5	532	23.79	6996	22.40	7528	22.50	
**Region**							
West Midlands	432	19.36	4861	15.56	5294	15.82	*p* = < 0.0001
North East	126	5.64	1126	3.60	1252	3.74	
North West	407	18.20	5958	19.07	6365	19.02	
Yorkshire and Humber	85	3.80	1084	3.47	1169	3.49	
East Midlands	58	2.59	744	2.38	802	2.40	
East of England	109	4.87	1617	5.18	1726	5.16	
London	258	11.54	5524	17.68	5782	17.28	
South East	466	20.84	6476	20.75	6942	20.74	
South West	294	13.15	3838	12.29	4132	12.35	
**Charlson group**							
0	1340	59.93	17,669	56.58	19,195	57.36	*p* = < 0.0001
1	85	3.80	1081	3.46	1151	3.44	
2	811	36.27	12,478	39.96	13,118	39.20	
**Age (years)**							
18–39	883	39.49	11,261	36.05	12,144	36.29	*p* < 0.0001
40–59	722	32.29	10,792	34.56	11,514	34.41	
60–79	592	26.48	7694	24.64	8286	24.76	
> 80	39	1.74	1481	4.74	1520	4.54	

### 3.2 Colectomy

The Kaplan‐ Meir cumulative incidence of colectomy at 1‐, 3‐, and 5‐ years following diagnosis was 2.59% (95% CI 2.42%–2.76%), 4.43% (95% CI 4.21%–4.65%) and 5.49% (95% CI 4.25%–5.75%) respectively (Figure S1). A new diagnosis of CRC was associated with 11.09% (248/2236) of colectomies performed in the cohort.

#### 3.2.1 Cumulative Incidence of Elective Colectomy

**FIGURE 2:**
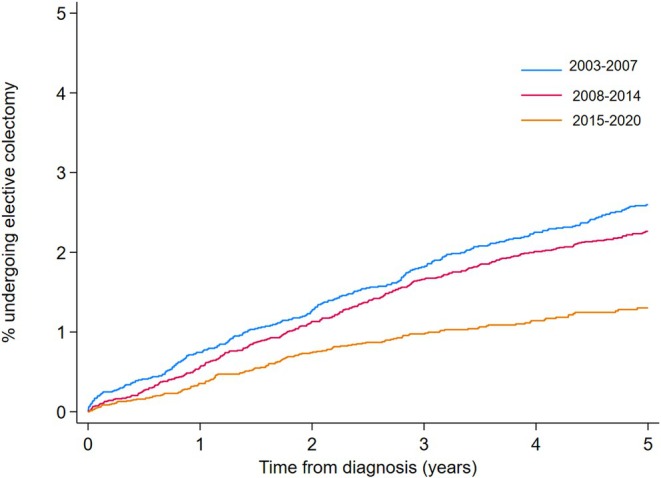
Cumulative incidence of elective colectomy. This graph demonstrates the cumulative incidence of elective colectomy across three time periods, calculated using Kaplan‐Meier survival methods. The curve shows the percentage of individuals who underwent elective colectomy from their date of diagnosis. Individuals were censored at the date of surgery, date of death, or date of last recorded data in CPRD. The x‐axis represents time in years from the date of diagnosis and the y‐axis represents the cumulative incidence of elective colectomy.

The cumulative incidence of elective surgery at 1‐, 3‐, and 5‐ years was 0.60% (95% CI 0.53–0.69%), 1.71% (95% CI 1.57–1.86%) and 2.40% (95% CI 2.23–2.58%) respectively. When comparing the incidence of colectomy over time, the 1‐year cumulative incidence of elective colectomy fell from 0.81% (95% CI 0.64–1.03%) to 0.42% (95% CI 0.32–0.55%) between 2003–2007 and 2015‐2020 time periods. The 5‐year incidence fell from 2.97% (95% CI 2.62%–3.36%) to 1.59% (95% CI 1.34–1.87%) for the corresponding time periods (Figure 2). There was a 53% reduction in the risk of elective colectomy for those diagnosed between 2015–2020 compared to 2003–2007 when accounting for other factors (aHR 0.47 (95% CI 0.38–0.58)). Females were 27% less likely to undergo elective colectomy compared to their male counterparts, aHR 0.73 (95% CI 0.64–0.83). Those aged 60–79 were 40% more likely to undergo colectomy (aHR 1.40 (95% CI 1.18–1.66)) and those over 80 years of age were 53% less likely (aHR 0.47 (95% CI 0.25–0.90)) to undergo elective colectomy compared to those aged 18–39 when adjusting for other variables. Those who had received a biologics or small molecules were 3 times as likely to need a colectomy, aHR 3.12 (95% CI 2.68–3.62) and those who had received an immunomodulator were twice as likely to undergo elective colectomy, aHR 2.60 (95% CI 2.24–3.02) (Table 2).

**TABLE 2:** Multivariate Cox regression model for risk of elective colectomy.

	Univariate			Multivariate		
	HR	*p*	95% CI	HR	*p*	95% CI
**Diagnosis year**						
2003–2007	ref	ref	ref	ref	ref	ref
2008–2014	0.81	< 0.01	0.70–0.93	0.77	< 0.01	0.67–0.89
2015–2020	0.49	< 0.01	0.40–0.60	0.47	< 0.01	0.38–0.58
**Gender**						
Male	ref	ref	ref	Ref	ref	ref
Female	0.69	< 0.01	0.61–0.78	0.73	< 0.01	0.64–0.83
**Diagnosis age**						
18–39	ref	ref	ref	Ref	ref	ref
40–59	0.85	0.03	0.73–0.98	0.97	0.70	0.83–1.12
60–79	1.08	0.28	0.93–1.27	1.40	< 0.01	1.18–1.66
> 80	0.28	< 0.01	0.15–0.53	0.47	0.02	0.25–0.90
**Charlson index**						
0	ref	ref	ref	Ref	ref	ref
1	1.21	0.25	0.87–1.66	1.24	0.19	0.89–1.73
2	0.98	0.78	0.86–1.18	0.97	0.72	0.85–1.12
**Biologics or small molecules**						
No	ref	ref	ref	Ref	ref	ref
Yes	4.64	< 0.01	4.06–5.29	3.18	< 0.01	2.68–3.62
**Steroids**						
No	ref	ref	ref	Ref	ref	ref
Yes	2.48	< 0.01	2.15–2.87	1.40	< 0.01	1.19–1.64
**Aminosalicylates**						
No	ref	ref	ref	Ref	ref	Ref
Yes	0.85	0.02	0.71–0.99	0.57	< 0.01	0.47–0.68
**Immunomodulators**						
No	ref	ref	ref	Ref	ref	Ref
Yes	4.08	< 0.01	3.60–4.63	2.60	< 0.01	2.23–3.02
**IMD**						
1	ref	ref	ref	Ref	ref	ref
2	1.06	0.59	0.85–1.32	1.03	0.76	0.82–1.30
3	1.15	0.19	0.93–1.41	1.07	0.51	0.87–1.33
4	1.13	0.24	0.92–1.40	1.06	0.56	0.85–1.33
5	1.28	0.01	1.05–1.57	1.20	0.96	0.96–1.50
**Region**						
South East	ref	ref	ref	Ref	ref	ref
North East	1.56	< 0.01	1.17–2.09	1.56	< 0.01	1.14–2.12
North West	0.91	0.37	0.75–1.12	0.85	0.16	0.68–1.06
Yorkshire and The Humber	1.34	0.07	0.97–1.83	1.24	0.18	0.90–1.73
East Midlands	0.88	0.60	0.56–1.39	0.86	0.52	0.54–1.36
West Midlands	1.32	< 0.01	1.09–1.60	1.21	0.06	0.99–1.49
East of England	0.83	0.28	0.60–1.15	0.96	0.82	0.68–1.34
London	0.63	< 0.01	0.50–0.80	0.68	< 0.01	0.54–0.87
South West	1.05	0.64	0.84–1.31	1.05	0.65	0.84–1.32

#### 3.2.2 Cumulative Incidence of Emergency Colectomy

**FIGURE 3:**
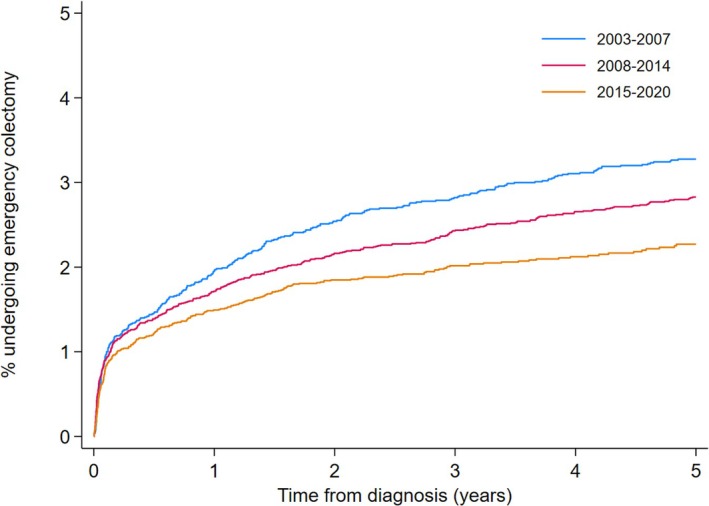
Cumulative incidence of emergency colectomy. This graph demonstrates the cumulative incidence of emergency colectomy across three time periods, calculated using Kaplan‐Meier survival methods. The curve shows the percentage of individuals who underwent emergency colectomy from their date of diagnosis. Individuals were censored at the date of surgery, date of death, or date of last recorded data in CPRD. The x‐axis represents time in years from the date of diagnosis, and the y‐axis represents the cumulative incidence of emergency colectomy.

The cumulative incidence of emergency surgery at 1‐, 3‐ and 5‐years was 2.00% (95% CI 1.86–2.16%), 2.81% (95% CI 2.64–3.00%) and 3.26% (95% CI 3.07–3.46%) respectively. When comparing the cumulative incidence of colectomy between different time periods, the 1‐year incidence fell from 2.32% (95% CI 2.02–2.66%) to 1.75% (95% CI 1.53–2.00%) between 2003–2007 and 2015–2020 and 5‐year incidence fell from 3.90% (95% CI 3.51%–4.34%) to 2.67% (95% CI 2.37–3.00%) (Figure 3). A reduction in emergency colectomy over time was confirmed on multivariate Cox‐regression. There was a 40% reduction in emergency colectomies in the 2015–2020 period compared to 2003–2007 when adjusting for co‐variates, aHR 0.60 (95% CI 0.51–0.70). As with elective colectomy, females were less likely to undergo emergency colectomy compared to males (aHR 0.71 (95% CI 0.63–0.80)). Age was associated with a reduction in emergency colectomy. Those aged 40–59 were 13% less likely, (aHR 0.87 (95% CI 0.76–0.99) and those over 80 years of age were 49% less likely, aHR 0.51 (95% CI 0.34–0.77) when compared to those aged 18–39 and accounting for other variables. Those that had previously received a biologic or small molecule were 4 times as likely to require an emergency colectomy, aHR 3.97 (95% CI 3.43–4.58) whereas those having received an immunomodulator were less likely though this failed to reach statistical significance on multivariate modelling, aHR 0.91 (95% CI 0.78–1.07) (Table 3).

**TABLE 3:** Multivariate Cox regression model for risk of emergency colectomy.

	Univariate			Multivariate		
	HR	*p*	95% CI	HR	*p*	95% CI
**Diagnosis year**						
2003–2007	ref	ref	ref	ref	ref	ref
2008–2014	0.86	0.03	0.76–0.98	0.88	0.09	0.77–1.02
2015–2020	0.68	< 0.01	0.58–0.79	0.60	< 0.01	0.51–0.70
**Gender**						
Male	ref	ref	ref	ref	Ref	ref
Female	0.71	< 0.01	0.64–0.80	0.71	< 0.01	0.63–0.80
**Diagnosis age**						
18–39	ref	ref	ref	Ref	ref	Ref
40–59	0.82	< 0.01	0.72–0.94	0.86	0.04	0.75–0.99
60–70	0.95	0.47	0.82–1.09	1.00	0.96	0.85–1.17
> 80	0.48	< 0.01	0.32–0.72	0.51	< 0.01	0.34–0.77
**Charlson index**						
0	ref	ref	ref	ref	ref	Ref
1	0.96	0.82	0.70–1.32	0.97	0.88	0.70–1.35
2	0.90	0.08	0.80–1.01	0.89	0.08	0.78–1.01
**Biologics or small molecules**						
No	ref	ref	ref	ref	ref	ref
Yes	3.27	< 0.01	2.88–3.71	3.96	< 0.01	3.43–4.58
**Steroids**						
No	ref	ref	ref	Ref	ref	Ref
Yes	1.03	0.61	0.92–1.15	1.03	0.62	0.91–1.17
**Aminosalicylates**						
No	ref	ref	ref	Ref	Ref	ref
Yes	0.26	< 0.01	0.23–0.29	0.22	< 0.01	0.20–0.25
**Immunomodualtors**						
No	ref	ref	ref	Ref	ref	Ref
Yes	1.26	< 0.01	1.04–1.44	0.84	0.08	0.78–1.07
**IMD**						
1	ref	ref	ref	Ref	ref	Ref
2	0.85	0.09	0.70–1.03	0.83	0.08	0.68–1.02
3	0.93	0.45	0.79–1.12	0.94	0.49	0.77–1.13
4	0.99	0.93	0.83–1.19	0.98	0.82	0.81–1.18
5	0.95	0.60	0.80–1.14	0.85	0.10	0.69–1.03
**Region**						
South East	ref	ref	ref	Ref	Ref	Ref
North East	1.51	< 0.01	1.16–1.98	1.43	0.12	1.08–1.91
North West	0.98	0.83	0.82–1.17	0.95	0.62	0.78–1.15
Yorkshire and The Humber	0.87	0.43	0.62–1.23	0.79	0.19	0.55–1.12
East Midlands	1.24	0.22	0.88–1.76	1.14	0.47	0.80–1.62
West Midlands	1.13	0.18	0.94–1.35	1.07	0.48	0.88–1.29
East of England	1.04	0.79	0.79–1.36	1.05	0.72	0.79–1.39
London	0.67	< 0.01	0.55–0.83	0.66	< 0.01	0.53–0.82
South West	1.06	0.51	0.87–1.30	1.06	0.59	0.86–1.29

### 3.3 90‐Day Post‐Operative Mortality

The overall 90‐day mortality rate following surgery was 2.75% (95% CI 2.17–3.49%). For elective surgery 90‐day mortality was 1.47% (95% CI 0.90–2.39%) and for emergency surgery 3.81% (95% CI 2.90–4.99%). In those less than 40 years of age the overall mortality was 0.25% (95% CI 0.06–1.00%) and was similar for those undergoing elective and emergency surgery, 0.29% (95% CI 0.04–2.08%) and 0.22% (95% CI 0.03–1.54%). 90‐day mortality was over three times higher following emergency colectomy than elective colectomy when accounting for other variables (aHR 2.83 (95% CI 1.59–5.02)). Age over 60 years was also associated with a significantly increased risk of mortality (Table S1).

### 3.4 Medications

#### 3.4.1 Steroids

One year following diagnosis the cumulative incidence of steroid prescription in general practice was 31.62% (95% CI 31.12–32.12%) and at 5 years it was 47.72% (95% CI 47.16–48.28). When comparing steroid use across different time periods, between 2003–2007 at 1‐year 33.78% (95% CI 32.78–34.80%) had received a steroid prescription which fell to 28.66% (95% CI 27.85–29.48%) for the 2015–2020 period. At 5‐years following diagnosis, the cumulative incidence of steroid prescriptions fell from 50.66% (95% CI 49.58–51.74%) to 43.45% (95% CI 42.46–44.46%) between the 2003–2007 to 2015–2020 periods (Table S2).

#### 3.4.2 Aminosalicylates

At one year following diagnosis, the cumulative incidence of aminosalicylate prescriptions in general practice was 76.67% (95% CI 76.20–77.12%) and at 5‐years was 84.36% (95% CI 83.95–84.76%). When comparing aminosalicylate prescriptions over the three time periods, aminosalicylate use increased from 70.59% (95% CI 69.61–71.56%) at 1‐year following diagnosis in 2003–2007 to 79.86% (95% CI 79.16–80.56%) in 2008‐2014 before falling again slightly in the 2015‐2020 period to 77.60% (95% CI 76.84–78.35%) (Table S3).

#### 3.4.3 Immunomodulators

The cumulative incidence of immunomodulator prescriptions in general practice at 1‐year was 9.57% (95% CI 9.26–9.90%) and by 5‐years was 19.15% (95% CI 18.71–19.60%).Immunomodulator prescriptions at 1‐year increased from 8.94% (95% CI 8.35–9.57%) between 2003–2007 to 10.79% (95% CI 10.26–11.35%) between 2008–2014 before falling again to 8.73% (95% CI 8.23–9.26%) in the 2015–2020 period (Table S4).

#### 3.4.4 Advanced Therapies

The incidence of advanced therapy use increased across the 3 time periods from 0.77% (95% CI 0.60–0.98%) at 1‐year for the 2003–2007 period to 7.10% (95% CI 6.65–7.58%) in the 2015–2020. At 5‐years following diagnosis, the cumulative incidence increased from 2.53% (95% CI 2.21–2.90%) in 2003‐2007 to 16.71% (95% CI 15.95–17.49%) in the 2015–2020 period (Table S5).

## 4 Discussion

The aim of this study was to describe trends in colectomy incidence and medication use following a new ulcerative colitis diagnosis. Cumulative colectomy rates were low, with elective colectomy halving over time and more modest declines in emergency colectomy, particularly in the first year post‐diagnosis. 90‐day mortality was low, though higher following emergency surgery and in older patients. General practitioner steroid prescriptions declined most after 2015, while aminosalicylate and immunomodulator prescriptions increased until 2015 before slightly declining as advanced therapy use rose substantially.

The smaller reduction in emergency colectomies may reflect a subset of patients with severe disease unresponsive to advanced therapies, making surgery unavoidable. However, some who might have previously required emergency colectomy may now be able to achieve sufficient disease control to delay to elective surgery with the use of advanced therapies and immunomodulators. Another factor might be an increase in the diagnosis of milder UC phenotypes with the introduction of faecal occult blood testing in 2006 for bowel cancer screening [31]. However, UK studies showing a decline in IBD incidence do not support this [32, 33]. Furthermore, a Canadian study by Bedi et al. reports that only 0.6% of patients who underwent colonoscopy following a positive faecal immunohistochemistry (FIT) were found to have colitis, with only 0.06% of the total screening population found to have UC [34].

Steroid prescriptions in the first year post‐diagnosis fell by only 5% despite improved access to specialist care [4] and are likely due to initial management implemented in primary care, where GPs commonly would prescribe steroids. The decline in immunomodulator and aminosalicylate use after 2015 coincides with NICE approval for biologics [29], suggesting a change in practice. We demonstrated a strong association between prior advanced therapy use and both elective and emergency colectomy, reflecting that advanced therapies are reserved for those with severe or refractory disease who have an inherent risk of requiring surgery. In contrast, those who had received an immunomodulator were more likely to undergo elective than emergency colectomy. This may reflect the use of immunomodulators in those less acutely unwell in whom elective decisions can be made.

Our study found a lower colectomy incidence after UC diagnosis than studies reported from Northern Europe [6, 35] and Canada [7, 36], possibly due to older cohorts utilised in those studies. Contemporary data from Switzerland [12] are more in keeping with colectomy rates reported from our cohort. Although we report rates higher than in Korea [5] and Southern Europe [35], these studies do not differentiate between elective and emergency colectomy, which represent different cohorts of patients.

Several UK studies have examined emergency colectomy trends. Similar to our study, Ahmed et al. analysed HES data; they found elective colectomy rates halved, but emergency rates showed no significant change [37] King et al. found that between 2007 and 2017, colectomy rates during acute admission for UC and at 12 months following admission fell from 8.7% to 6.2% and from 6% to 4.6% respectively [38] and Shawhidi et al. reported that colectomy for UC‐specific emergency admissions reduced from 9.4% to 7.3% and a 3% year‐onon‐year decline in unplanned colectomies between 2005 and 2014 [39]. Interestingly, Worley et al. observed a 17% reduction in colectomy incidence at 1 year following an acute admission for UC when comparing 2003–2007 with 2013–2016. It is important to note that Ahmad et al. calculated colectomy rates as a proportion of admissions, which are likely to have increased over time due to admission for biological therapy. These hospital‐based studies define cohorts from hospital admissions which represent a group of patients with disease severity sufficient to necessitate hospitalisation, whereas our study provides an inclusive picture of the disease spectrum from diagnosis.

In our study, 54.42% of colectomies were unplanned, which is slightly higher than that reported from comparable healthcare settings. Another English study reported a 44% emergency colectomy rate [40] and 41.39% has been reported in an Australian study [41]. The higher proportion may reflect our more recent study period, which includes the COVID‐19 pandemic and the doubling of NHS elective waiting lists [42]. We report that 11.08% of colectomies in patients with UC followed a CRC diagnosis, which aligns with other published studies [41, 43].

International studies have failed to demonstrate a significant reduction in colectomy rates in the post‐biologic era. An Italian study by Aratari et al. found infliximab did not reduce long‐term colectomy risk in those hospitalised with acute severe UC [44]. Zhao et al., using population‐based data from Norway, Sweden, and Denmark, reported stable surgery rates in Scandinavia despite increasing biologic use [45]. Using population‐level data from the U.S., Kayal et al. observed a reduction in emergent colectomy, though the overall burden of surgery remained stable, suggesting a shift from emergency to elective procedures [46]. Surprisingly, Kin et al., on analysing private insurance claims, reported colectomy rates increasing post‐2007 [47]. The observed differences may be due to our use of a more contemporary cohort and a more representative population by incorporating both primary and secondary care data to establish an incidence cohort from diagnosis.

When considering prescription rates for the treatment of UC, our findings align with existing studies in the literature. A Japanese study, as with our study, found the prescription rate of aminosalicylates dropped by 6% towards the end of the study period in 2018 [48]. US administrative claims data found that in the period 2008–2015, 6% of people with UC received a biologic [49]. A Korean study using an incident cohort found that by 2015, 13.4% had received a biologic [50] and a Scandinavian study reported that in 2015, in Denmark, 16% of patients had received a biologic and 18% had in Norway [45].

There are several limitations to our study which should be considered. Whilst we accurately report post‐operative mortality, limited data prevented robust reporting of post‐operative complications. Major post‐operative complications occur in approximately 10% of UC colectomies [51, 52, 53, 54] which may significantly impact recovery and quality of life and would be an important consideration for anyone undergoing colectomy. Furthermore, we included all types of colectomy in our analysis (i.e., those with and without anastomoses). Although our primary aim was to evaluate general trends, this heterogeneity limits the ability to draw conclusions about the safety of specific surgeries which have different risk profiles. We have only been able to capture steroid, aminosalicylate, and immunomodulator prescriptions in primary care. Although a number of prescriptions will be initiated and administered in secondary care, in England it is common for these to be continued in primary care, particularly for long‐term treatments due to shared care models which integrate prescribing responsibilities between specialists and general practitioners. We have analysed our data by censoring patients after the first prescription, so this issue is unlikely to distort the overall picture. Although our data does not capture subcutaneous and oral advanced therapy prescriptions administered in secondary care, the marked increase in IV biologic and prescription of advanced therapies in primary care over the study period serves as a proxy for the growing uptake of advanced therapies; it is likely that the trend in IV biologic use mirrors that of all advanced therapies. Despite this limitation meaning that our absolute estimate of advanced therapy use is likely to understate the true level, our study still demonstrates a temporal association between increased advanced therapy use and declining colectomy rates, reflecting the broad impact of advanced therapies at a population level.

We acknowledge that our median age at diagnosis, 47 years old, is higher than that reported in other population‐based studies [55]. This is largely explained by the exclusion of paediatric cases with no upper age limit for inclusion, resulting in a median age that reflects distribution within an adult cohort rather than the entire population. However, it may in part reflect the increasing incidence of elderly‐onset UC, as reported in other published population‐based studies [56, 57, 58]. To our knowledge, this is the largest and most contemporary incident cohort study of UC in England that can combine both primary and secondary care data. It spans a 17‐year period with a median follow‐up time of 8 years, giving a longitudinal view of how the management of UC has changed and a more comprehensive understanding of long‐term outcomes and disease progression.

## 5 Conclusion

This paper describes the natural history of ulcerative colitis with regard to the medical and surgical management. Although causality cannot be attributed, we have demonstrated that a decreased incidence of colectomy and immunomodulator use coincides with the increased use of advanced therapies. In the context of other published literature, it seems most plausible that this is a consequence of multi‐factorial improvements in medical care. Post‐operative mortality is low and this should be reassuring for those failing to respond to medical management or developing disease‐related complications requiring surgical management. Research encompassing a wider range of advanced therapies, post‐operative complications, and long‐term quality of life in medically and surgically treated patients will enhance the evidence base for shared decision making between clinicians and patients. In the absence of such comprehensive data, our findings provide valuable insights to support such discussions.

## Author Contributions

**J. Couch:** conceptualization, methodology, formal analysis, writing – original draft, investigation. **K. Thomas:** supervision, writing – review and editing. **T. R. Card:** methodology, writing – review and editing, supervision. **D. J. Humes:** formal analysis, writing – review and editing, visualization, methodology, conceptualization, supervision.

## Ethics Statement

The study had approval from the Independent Scientific Advisory Committee approval board. This study involved the use of anonymised primary care and linked data routinely available via CPRD and therefore separate research ethics approval was not required.

## Conflicts of Interest

The authors declare no conflicts of interest.

## Supporting information


**Data S1:** apt70319‐sup‐0001‐supinfo.docx.

**Appendix S1:** apt70319‐sup‐0002‐AppendixS1.docx.

## Data Availability

The data that support the findings of this study are available from CPRD but restrictions apply to the availability of these data, which were used under licence for this study and are not publicly available. Data are, however, available from the authors upon reasonable request and with permission of CPRD.
