# Supplementary material for: Temporal Trends in Medical and Surgical Management of Ulcerative Colitis in England: 2003–2020
Source: Aliment Pharmacol Ther. 2025 Aug 8;63(1):119–31. doi: 10.1111/apt.70319 (PMC12690229; doi:10.1111/apt.70319)
Supplement: Supplementary file 1 — Data S1: apt70319‐sup‐0001‐supinfo.docx. [file APT-63-119-s001.docx]

**Figure 1: Cumulative incidence of colectomy**

**
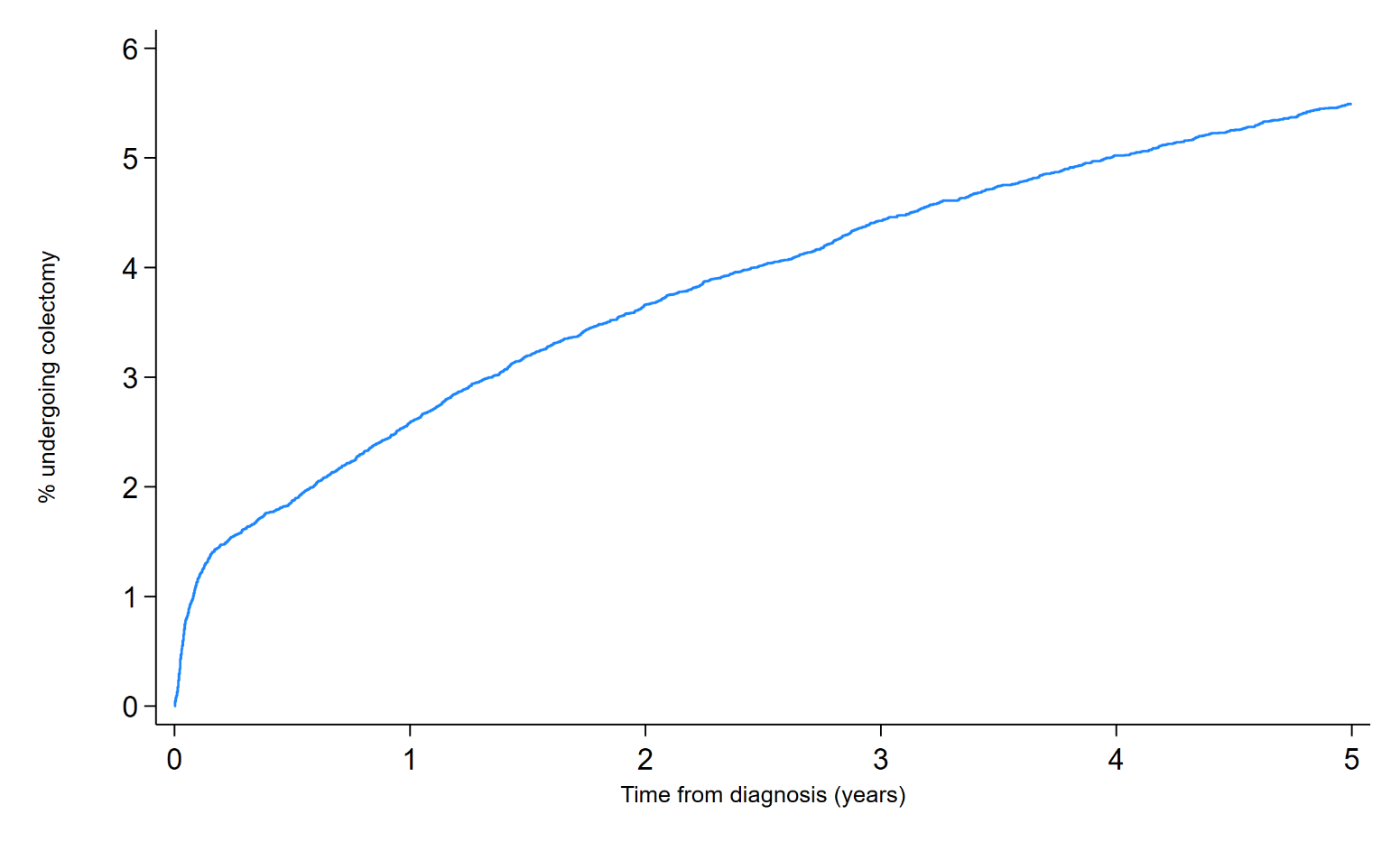
**

**Figure 1:** Cumulative incidence of colectomy. This graph demonstrates the cumulative incidence of colectomy over the entire follow-up period, calculated using Kaplan-Meier survival methods. The curve shows the percentage of individuals who underwent colectomy from their date of diagnosis. Individuals were censored at the date of surgery, date of death or date of last recorded data in CPRD. The x-axis represents time in years from the date of diagnosis and the y-axis represents the cumulative incidence of colectomy.

**Table 1: Multivariate Cox regression model for risk of 90-day mortality following colectomy**

|  | Univariate | | |  | Multivariate | | |
| --- | --- | --- | --- | --- | --- | --- | --- |
|  | **HR** | **p-value** | **95% CI** |  | **HR** | **p-value** | **95% CI** |
| Admission Type |  |  |  |  |  |  |  |
| Elective | ref | ref | ref |  | ref | ref | ref |
| Emergency | 2.61 | <0.01 | 1.49-4.59 |  | 2.83 | <0.001 | 1.59-5.01 |
| Gender |  |  |  |  |  |  |  |
| Male | ref | ref | ref |  | ref | ref | ref |
| Female | 0.76 | 0.28 | 0.46-1.25 |  | 0.81 | 0.41 | 0.48-1.34 |
| Age at surgery |  |  |  |  |  |  |  |
| 18-39 | ref | ref | ref |  | ref | ref | ref |
| 40-59 | 2.06 | 0.40 | 0.37-11.26 |  | 2.09 | 0.39 | 0.38-11.45 |
| 69-79 | 27.18 | <0.01 | 6.60-113.82 |  | 30.01 | <0.01 | 7.22-124.57 |
| >80 | 52.15 | <0.01 | 11.67-233.02 |  | 54.40 | <0.1 | 11.96-247.39 |
| Year of surgery |  |  |  |  |  |  |  |
| 2003-2007 | ref | ref | ref |  | ref | ref | ref |
| 2008-2014 | 0.69 | 0.24 | 0.38-1.27 |  | 0.82 | 0.52 | 0.43-1.51 |
| 2015-2020 | 0.62 | 0.14 | 0.33-1.16 |  | 0.72 | 0.33 | 0.36-1.40 |
| Charlson Index |  |  |  |  |  |  |  |
| 0 | ref | ref | ref |  | ref | ref | ref |
| 1 | 0.57 | 0.58 | 0.07-4.20 |  | 0.28 | 0.33 | 0.03-2.09 |
| 2 | 2.42 | <0.01 | 1.47-3.99 |  | 1.43 | 0.17 | 0.86-2.38 |
| Minimally invasive surgery |  |  |  |  |  |  |  |
| No | ref | ref | ref |  | ref | ref | ref |
| Yes | 0.42 | 0.009 | 0.22-0.81 |  | 0.79 | 0.50 | 0.40-1.56 |

**Table 2: Cumulative incidence of steroid prescriptions**

|  | Year of diagnosis | | |
| --- | --- | --- | --- |
| Time from diagnosis (years) | **2003-2007** | **2008-2015** | **2015-2020** |
| 1 | 0.34 (0.33-0.35) | 0.33 (0.33-0.34) | 0.29 (0.28-0.29) |
| 2 | 0.40 (0.39-0.41) | 0.40(0.39-0.41) | 0.35 (0.34-0.36) |
| 3 | 0.45 (0.44-0.46) | 0.44 (0.43-0.45) | 0.39 (0.38-0.40) |
| 4 | 0.48 (0.47-0.49) | 0.47 (0.46-0.48) | 0.41 (0.40-0.42) |
| 5 | 0.51 (0.50-0.52) | 0.49 (0.48-0.50) | 0.43 (0.42-0.44) |

**Table 3: Cumulative incidence of aminosalicylate prescriptions**

|  | Year of diagnosis | | |
| --- | --- | --- | --- |
| Time from diagnosis (years) | **2003-2007** | **2008-2015** | **2015-2020** |
| 1 | 0.71 (0.70-0.72) | 0.80 (0.79-0.80) | 0.78 (0.77-0.78) |
| 2 | 0.75 (0.41-0.76) | 0.83 (0.83-0.84) | 0.81 (0.80-0.82) |
| 3 | 0.77 (0.76-0.78) | 0.85 (0.84-0.86) | 0.83 (0.82-0.83) |
| 4 | 0.78 (0.78-0.79) | 0.86 (0.86-0.87) | 0.84 (0.83-0.84) |
| 5 | 0.80 (0.79-0.80) | 0.87 (0.86-0.87) | 0.84 (0.84-0.85) |

**Table 4: Cumulative incidence of immunomodulator prescriptions**

|  | Year of diagnosis | | |
| --- | --- | --- | --- |
| Time from diagnosis (years) | **2003-2007** | **2008-2015** | **2015-2020** |
| 1 | 0.09 (0.28-0.10) | 0.11 (0.10-0.11) | 0.09 (0.08-0.09) |
| 2 | 0.13 (0.10-0.11) | 0.15 (0.15-0.16) | 0.13 (0.13-0.14) |
| 3 | 0.16 (0.15-0.16) | 0.18 (0.17-0.19) | 0.16 (0.15-0.16) |
| 4 | 0.17 (0.16-0.18) | 0.19 (0.19-0.20) | 0.17 (0.16-0.18) |
| 5 | 0.18 (0.18-0.19) | 0.20 (0.20-0.21) | 0.18 (0.17-0.18) |

**Table 5: Cumulative incidence of advanced therapies**

|  | Year of diagnosis | | |
| --- | --- | --- | --- |
|  | **2003-2007** | **2008-2015** | **2015-2020** |
| Time from diagnosis (years) | **Cumulative incidence (95% CI)** | | |
| 1 | 0.001 (0.005-0.008) | 0.02 (0.018-0.024) | 0.07 (0.06-0.08) |
| 2 | 0.01 (0.009-0.013) | 0.03 (0.02-0.04) | 0.10 (0.09-0.11) |
| 3 | 0.02 (0.018-0.023) | 0.05 (0.05-0.05) | 0.13(0.12-0.14) |
| 4 | 0.02 (0.018-0.025) | 0.06 (0.05-0.06) | 0.15(0.14-0.16) |
| 5 | 0.02 (0.02-0.03) | 0.07 (0.06-0.07) | 0.17(0.15-0.17) |
