# Supplementary material for: Temporal Trends in Medical and Surgical Management of Ulcerative Colitis in England: 2003–2020
Source: Aliment Pharmacol Ther. 2025 Aug 8;63(1):119–31. doi: 10.1111/apt.70319 (PMC12690229; doi:10.1111/apt.70319)
Supplement: Supplementary file 2 — Appendix S1: apt70319‐sup‐0002‐AppendixS1.docx. [file APT-63-119-s002.docx]

## Appendix 1 – codes for identification of inflammatory bowel disease diagnoses

### Inflammatory bowel disease medcodes for CPRD

**Medcodeid Term**

4411741000006119 Acute ulcerative colitis

14195011000006120 Acute ulcerative pancolitis

309743013 Arthropathy in ulcerative colitis

7294371000006118 Chronic ulcerative colitis

7260561000006112 Chronic ulcerative pancolitis

3346671000006113 Chronic ulcerative proctitis

3351321000006113 Chronic ulcerative proctosigmoiditis

2532953017 Exacerbation of ulcerative colitis

411543016 H/O: ulcerative colitis

5574431000006111 History of ulcerative colitis

309836013 Juvenile arthritis in ulcerative colitis

1816421000006112 Left sided ulcerative colitis

3774141000006118 Mild chronic ulcerative colitis

2726621000006117 Severe chronic ulcerative colitis

3553391000006113 UC - Ulcerative colitis

3346681000006111 UC - Ulcerative colitis confined to rectum

85901000006116 Ulcerative (chronic) ileocolitis

107644019 Ulcerative colitis

85931000006112 Ulcerative colitis and/or proctitis

3346691000006114 Ulcerative colitis confined to rectum

3351341000006118 Ulcerative colitis confined to rectum and sigmoid colon

7502561000006118 Ulcerative colitis in remission

85891000006115 Ulcerative enterocolitis

302953018 Ulcerative ileocolitis

2872721013 Ulcerative pancolitis

496249010 Ulcerative proctitis

435370011 Ulcerative proctocolitis

302956014 Ulcerative proctocolitis NOS

3351351000006116 Ulcerative proctosigmoiditis

496332018 Ulcerative rectosigmoiditis

906191000006113 [RFC] Ulcerative colitis

303762015 [X]Other ulcerative colitis

3351311000006117 Chronic ulcerative rectosigmoiditis

3553381000006110 Colitis gravis

8463921000006112 Abscess of intestine due to Crohn's disease

4808981000006112 Arthropathy in Crohn disease

309744019 Arthropathy in Crohn's disease

601031000006119 CC - Crohn's colitis

3047391000006119 CD - Crohn's disease

5548931000006111 CDAI - Crohn's disease activity index

3047411000006119 Crohn disease

7060771000006117 Crohn disease in remission

3113551000006113 Crohn disease of ileum

2621151000006116 Crohn disease of large bowel

4785581000006112 Crohn disease of terminal ileum

6664411000006115 Crohn disease of vulva

6839131000006116 Crohn stricture of colon

56765016 Crohn's disease

1222351011 Crohn's disease NOS

5548921000006113 Crohn's disease activity index

7060761000006112 Crohn's disease in remission

3316751000006117 Crohn's disease of colon

3414681000006119 Crohn's disease of duodenum

302940016 Crohn's disease of ileum

396357012 Crohn's disease of large bowel

4784091000006115 Crohn's disease of oral soft tissues

2559781000006115 Crohn's disease of rectum

3668441000006118 Crohn's disease of small AND large intestines

302941017 Crohn's disease of small intestine

601091000006115 Crohn's disease of terminal ileum

302939018 Crohn's disease of the ileum unspecified

7988841000006110 Crohn's disease with arthritis

3113541000006111 Crohn's ileitis

2559801000006116 Crohn's proctitis

179501000006113 Crohn's regional enteritis

6839121000006119 Crohn's stricture of colon

3047421000006110 Crohns disease

3316811000006110 Crohns disease, colon

2621161000006119 Crohns disease, large intestine

3420891000006119 Crohns disease, small intestine

6853111000006114 Exacerbation of Crohn disease of large intestine

6853131000006115 Exacerbation of Crohn disease of small intestine

2532950019 Exacerbation of Crohn's disease of large intestine

2532958014 Exacerbation of Crohn's disease of small intestine

3316791000006111 GC - granulomatous colitis

6582841000006118 Gastrointestinal Crohn's disease

3316771000006110 Granulomatous colitis

56770011 Granulomatous enteritis

8035641000006112 History of Crohns disease

4809351000006111 Juvenile arthritis in Crohn disease

309833017 Juvenile arthritis in Crohn's disease

4784111000006112 Oral Crohn's disease

302322010 Orofacial Crohn's disease

5085631000006115 Perianal Crohn disease

5085621000006118 Perianal Crohn's disease

7959171000006110 Perianal fistula due to Crohn's disease

3047431000006113 RE - regional enteritis

886291000006112 Regional enteritis - Crohn

179521000006115 Regional enteritis of the colon

497569010 Regional enteritis of the duodenum

1495442018 Regional enteritis of the large bowel

488238014 Regional enteritis of the rectum

179571000006119 Regional enteritis of the small bowel

302946010 Regional ileocolitis

6664401000006118 Vulval Crohn's disease

906051000006118 [RFC] Crohns disease

303761010 [X]Other Crohn's disease

3316801000006112 crohn disease of colon

3414711000006118 crohn disease of duodenum

2559821000006114 crohn disease of rectum

3420881000006117 crohn disease of small bowel

3414701000006116 crohn's duodenitis

3047381000006117 granulomatous enteritis

5551391000006118 harvey bradshaw index

3316761000006115 regional colitis

302947018 regional enteritis NOS

302937016 regional enteritis of jejunum

2559791000006117 regional enteritis of rectum

3420851000006113 regional enteritis of the small bowel

2338581000000110 Dietary education for inflammatory bowel disease

7489581000006113 History of inflammatory bowel disease

2891431000006118 IBD - Inflammatory bowel disease

41137017 Inflammatory bowel disease

2269901000000115 Management of IBD (inflammatory bowel disease)

2269891000000116 Management of inflammatory bowel disease

353407019 Indeterminate colitis

3047371000006115 regional enteritis

3420861000006110 regional ileitis of small intestine

## Inflammatory bowel disease medcodes for HES

**ICD-10 code Term**

K50 Crohn disease [regional enteritis]

K50.0 Crohn disease of small intestine

K50.1 Crohn disease of large intestine

k50.8 Other Crohn disease

K50.9 Crohn disease, unspecified

K51 Ulcerative colitis

K51.0 Ulcerative (chronic) pancolitis

K51.2 Ulcerative (chronic) proctitis

K51.3 Ulcerative (chronic) rectosigmoiditis

K51.5 Left sided colitis

K18 Other ulcerative colitis

K51.9 Ulcerative colitis, unspecified

K52.3 Indeterminate colitis

## OPCS-4 codes for colectomy

**OPCS-4 Procedure**

H05.1 Total colectomy and anastomosis of ileum to rectum

H05.2 Total colectomy and ileostomy and creation of rectal fistula hfq

H05.3 Total colectomy and ileostomy nec

H05.8 Other specified subtotal excision of colon

H05.9 Unspecified subtotal excision of colon

H04.1 Panproctocolectomy and ileostomy Includes: Proctocolectomy nec

H04.2 Panproctocolectomy and anastomosis of ileum to anus and creation of pouch hfq

H04.3 Panproctocolectomy and anastomosis of ileum to anus nec

H06 Extended excision of right hemicolon Includes: Excision of right colon and other segment of ileum or colon and surrounding tissue. Caecum

H06.1 Extended right hemicolectomy and end to end anastomosis

H06.2 Extended right hemicolectomy and anastomosis of ileum to colon

H06.3 Extended right hemicolectomy and anastomosis nec

H06.4 Extended right hemicolectomy and ileostomy hfq

H06.5 Extended right hemicolectomy and end to side anastomosis

H06.8 Other specified extended excision of right colon

H06.9 Unspecified extended excision of right colon

H08 Excision of transverse colon

H08.1 Transverse colectomy and end to end anastomosis

H08.2 Transverse colectomy and anastomosis of ileum to colon

H08.3 Transverse colectomy and anastomosis NEC

H08.4 Transverse colectomy and ileostomy HFQ

H08.5 Transverse colectomy and exteriorisation of bowel NEC

H08.6 Transverse colectomy and end to side anastomosis

H08.8 Other specified

H08.9 Unspecified

H09 Excision of left hemicolon

H09.1 Left hemicolectomy and end to end anastomosis of colon to rectum

H09.2 Left hemicolectomy and end to end anastomosis of colon to colon

H09.3 Left hemicolectomy and anastomosis NEC

H09.4 Left hemicolectomy and ileostomy HFQ

H09.5 Left hemicolectomy and exteriorisation of bowel NEC

H09.6 Left hemicolectomy and end to side anastomosis

H09.8 Other specified

H09.9 Unspecified

H10 Excision of sigmoid colon

H10.1 Sigmoid colectomy and end to end anastomosis of ileum to rectum

H10.2 Sigmoid colectomy and anastomosis of colon to rectum

H10.3 Sigmoid colectomy and anastomosis NEC

H10.4 Sigmoid colectomy and ileostomy NFQ

H10.5 Sigmoid colectomy and exteriorisation of bowel NEC

H10.6 Sigmoid colectomy and end to side anastomosis

H10.8 Other specified

H10.9 Unspecified

H11 Other excision of colon Includes: Excision of colon where segment removed is not stated

H11.1 Colectomy and end to end anastomosis of colon to colon NEC

H11.2 Colectomy and side to side anastomosis of ileum to colon NEC

H11.3 Colectomy and anastomosis NEC

H11.4 Colectomy and ileostomy NEC

H11.5 Colectomy and exteriorisation of bowel NEC

H11.6 Colectomy and end to side anastomosis NEC

H11.8 Other specified

H11.9 Unspecified, Includes: Colectomy NEC, Hemicolectomy NEC

H07.1 Right hemicolectomy and end to end anastomosis of ileum to colon

H07.2 Right hemicolectomy and side to side anastomosis of ileum to transverse colon

H07.3 Right hemicolectomy and anastomosis NEC

H07.4 Right hemicolectomy and ileostomy HFQ

H07.5 Right hemicolectomy and end to side anastomosis

H07.8 Other specified other excision of right hemicolon

H07.9 Unspecified other excision of right hemicolon

H29.1 Subtotal excision of colon and rectum and creation of colonic pouch and anastomosis of colon to anus

H29.2 Subtotal excision of colon and rectum and creation of colonic pouch NEC

H29.3 Subtotal excision of colon and creation of colonic pouch and anastomosis of colon to rectum

H29.4 Subtotal excision of colon and creation of colonic pouch NEC

H29.8 Other specified subtotal excision of colon

H29.9 Unspecified subtotal excision of colon

## Inflammatory bowel disease medications

## Infusion of biologics

**OPCS Infusion**

x298 Intermittent intravenous infusion of therapeutic substance

x281 Intermittent intravenous infusion of therapeutic substance

x282 Intermittent subcutaneous infusion of therapeutic substance

x288 Other specified intermittent infusion of therapeutic substance

x289 Unspecified intermittent infusion of therapeutic substance

x293 Continuous subcutaneous infusion of therapeutic substance NEC

x299 Unspecified continuous Infusion of therapeutic substance

x891 Monoclonal antibodies Band 1

x921 Cytokine inhibitor drugs Band 1

x292 Continuous intravenous infusion of therapeutic substance NEC

## Steroids

**Prodcodeid Product name**

143041000033117 Betnelan 500microgram tablets

144641000033113 Betamethasone 500microgram soluble tablets sugar free

144841000033114 Betnesol 500microgram soluble tablets

145441000033110 Betamethasone 500microgram tablets

169341000033114 Budesonide 3mg gastro-resistant modified-release capsules

370241000033111 Co-triamterzide 50mg/25mg tablets

430841000033118 Decadron 500microgram tablets

431541000033114 Deltacortril 2.5mg gastro-resistant tablets

431641000033110 Deltacortril 5mg gastro-resistant tablets

431841000033111 Dexamethasone 2mg tablets

431941000033115 Dexamethasone 500microgram tablets

521541000033116 Entocort CR 3mg capsules

740241000033111 Hydrocortisone 10mg/5ml oral suspension

742241000033110 Hydrocortisone 20mg tablets

744541000033116 Hydrocortisone 10mg tablets

744641000033115 Hydrocortone 10mg tablets

744741000033112 Hydrocortone 20mg tablets

898441000033115 Medrone 100mg tablets

898541000033119 Methylprednisolone 100mg tablets

899641000033110 Medrone 16mg tablets

899741000033118 Medrone 2mg tablets

899841000033111 Medrone 4mg tablets

903141000033113 Methylprednisolone 16mg tablets

904141000033111 Methylprednisolone 2mg tablets

904241000033116 Methylprednisolone 4mg tablets

1114241000033116 Prednisolone 2.5mg gastro-resistant tablets

1114341000033114 Prednisolone 5mg gastro-resistant tablets

1127341000033111 Prednisolone 5mg soluble tablets

1130741000033110 Prednisolone 25mg tablets

1131741000033117 Prednisolone 1mg tablets

1131841000033110 Prednisolone 2.5mg tablets

1131941000033119 Prednisolone 5mg tablets

1718241000033117 Budenofalk 3mg gastro-resistant capsules

1718341000033110 Budesonide 3mg gastro-resistant capsules

1830141000033115 Hydrocortisone 5mg/5ml oral suspension

2146441000033119 Dexsol 2mg/5ml oral solution

2146541000033118 Dexamethasone 2mg/5ml oral solution sugar free

2149441000033113 Hydrocortisone 25mg/5ml oral suspension

2623741000033116 Hydrocortisone 5mg/5ml oral suspension sugar free

4214041000033115 Beclometasone 5mg gastro-resistant modified-release tablets

4214141000033116 Clipper 5mg gastro-resistant modified-release tablets

5506241000033117 Dexamethasone 4mg tablets

5900641000033111 Dexamethasone 5mg/5ml oral solution

5900741000033119 Dexamethasone 5mg/5ml oral suspension

5900841000033112 Dexamethasone 500micrograms/5ml oral solution

5900941000033116 Dexamethasone 500micrograms/5ml oral suspension

5990141000033115 Prednisone 1mg modified-release tablets

5990241000033110 Prednisone 2mg modified-release tablets

5990341000033117 Prednisone 5mg modified-release tablets

5990641000033113 Lodotra 1mg modified-release tablets

5990741000033116 Lodotra 2mg modified-release tablets

5990841000033114 Lodotra 5mg modified-release tablets

6512041000033112 Budesonide 9mg gastro-resistant granules sachets

6512141000033111 Budenofalk 9mg gastro-resistant granules sachets

6512741000033110 Martapan 2mg/5ml oral solution

8141941000033110 Hydrocortisone 5mg modified-release tablets

8142041000033116 Hydrocortisone 20mg modified-release tablets

8494441000033110 Dilacort 2.5mg gastro-resistant tablets

8494541000033111 Dilacort 5mg gastro-resistant tablets

9119141000033118 Prednisolone 15mg/5ml oral solution

9122441000033114 Dexamethasone 10mg/5ml oral solution sugar free

10212141000033112 Prednisolone 10mg tablets

10212341000033114 Prednisolone 20mg tablets

10212441000033116 Pevanti 2.5mg tablets

10212541000033120 Pevanti 5mg tablets

10212641000033118 Pevanti 10mg tablets

10212741000033110 Pevanti 20mg tablets

10212941000033112 Pevanti 25mg tablets

10213841000033110 Dexamethasone 20mg/5ml oral solution sugar free

10262941000033112 Budesonide 9mg modified-release tablets

10263141000033116 Cortiment 9mg modified-release tablets

10266841000033116 Prednisolone 5mg/5ml oral solution unit dose

10494341000033116 Prednisolone 10mg/ml oral solution sugar free

11072141000033116 Dexamethasone 2mg soluble tablets sugar free

11072241000033110 Dexamethasone 4mg soluble tablets sugar free

11072341000033116 Dexamethasone 8mg soluble tablets sugar free

11507841000033116 Prednisolone 1mg gastro-resistant tablets

11664841000033116 Prednisolone 30mg tablets

12389541000033112 Glensoludex 2mg soluble tablets

12389641000033114 Glensoludex 4mg soluble tablets

12389741000033116 Glensoludex 8mg soluble tablets

12398241000033120 Dexamethasone 40mg tablets

12575941000033114 Dexamethasone 8mg tablets

12633241000033112 Hydrocortisone 500microgram granules in capsules for opening

12633341000033118 Hydrocortisone 1mg granules in capsules for opening

12633441000033112 Hydrocortisone 2mg granules in capsules for opening

12633541000033112 Hydrocortisone 5mg granules in capsules for opening

12633641000033114 Alkindi 0.5mg granules in capsules for opening

12633741000033116 Alkindi 1mg granules in capsules for opening

12633841000033110 Alkindi 2mg granules in capsules for opening

12633941000033120 Alkindi 5mg granules in capsules for opening

12641241000033112 Budesonide 1mg orodispersible tablets sugar free

12890041000033114 Hydrocortisone 10mg soluble tablets sugar free

13010241000033114 Hydventia 10mg tablets

13010341000033116 Hydventia 20mg tablets

13897441000033114 Hydrocortisone 2.5mg tablets

13897541000033110 Hydrocortisone 5mg tablets

13897641000033112 Hydrocortisone 15mg tablets

13915541000033112 Hydrocortisone 5mg modified-release capsules

13915641000033112 Hydrocortisone 10mg modified-release capsules

13948241000033112 Dexamethasone 10mg soluble tablets sugar free

13948341000033120 Dexamethasone 20mg soluble tablets sugar free

14025241000033114 Hydrocortisone 5mg dispersible tablets sugar free

14025341000033116 Hydrocortisone 10mg dispersible tablets sugar free

14043541000033112 Budesonide 500microgram orodispersible tablets sugar free

## Aminosalicylates

**Prodcodeid Product name**

83441000033112 Asacol 1g/application foam enema

85441000033113 Asacol 250mg suppositories

85541000033114 Asacol 500mg suppositories

439141000033116 Dipentum 250mg capsules

463341000033113 Dipentum 500mg tablets

875441000033115 Mesalazine 250mg gastro-resistant tablets

875641000033118 Mesalazine 2g/59ml enema

875741000033110 Mesalazine 1g/100ml enema

876141000033116 Mesalazine 1g/application foam enema

888341000033110 Mesalazine 500mg modified-release tablets

892541000033117 Mesalazine 1g suppositories

893341000033116 Mesalazine 250mg suppositories

893441000033110 Mesalazine 500mg suppositories

1003241000033110 Olsalazine 250mg capsules

1003541000033112 Olsalazine 500mg tablets

1054841000033118 Pentasa 500mg modified-release tablets

1062141000033111 Pentasa Mesalazine 1g/100ml enema

1063041000033115 Pentasa 1g suppositories

1236641000033117 Salofalk 250mg gastro-resistant tablets

1237441000033116 Salazopyrin EN-Tabs 500mg

1237541000033115 Salofalk 2g/59ml enema

1256241000033119 Salazopyrin 250mg/5ml oral suspension

1256341000033112 Salofalk 500mg suppositories

1256841000033115 Salazopyrin 500mg suppositories

1257341000033114 Salazopyrin 500mg tablets

1655641000033114 Mesalazine 400mg gastro-resistant tablets

1902541000033110 Mesalazine 1g modified-release granules sachets sugar free

1902641000033111 Pentasa 1g modified-release granules sachets

2770741000033117 Salofalk 1g/application foam enema

2799341000033117 Ipocol 400mg gastro-resistant tablets

2980741000033117 Asacol 400mg MR gastro-resistant tablets

3005841000033116 Mesalazine 1g gastro-resistant modified-release granules sachets sugar free

3005941000033112 Mesalazine 500mg gastro-resistant modified-release granules sachets sugar free

3006041000033119 Salofalk 1g gastro-resistant modified-release granules sachets

3006141000033115 Salofalk 500mg gastro-resistant modified-release granules sachets

3014141000033112 Mesren MR 400mg gastro-resistant tablets

3103941000033119 Sulfasalazine 500mg gastro-resistant tablets

3104141000033118 Sulfasalazine 500mg suppositories

3104341000033115 Sulfasalazine 250mg/5ml oral suspension

3104541000033110 Sulfasalazine 500mg tablets

4323741000033116 Mesalazine 1.2g gastro-resistant modified-release tablets

4324441000033113 Mezavant XL 1200mg tablets

4425841000033118 Mesalazine 800mg gastro-resistant tablets

4425941000033114 Asacol 800mg MR gastro-resistant tablets

4522641000033119 Mesalazine 2g modified-release granules sachets sugar free

4522741000033111 Pentasa 2g modified-release granules sachets

4804141000033111 Mesalazine 1.5g gastro-resistant modified-release granules sachets sugar free

4804241000033116 Salofalk 1.5g gastro-resistant modified-release granules sachets

4816641000033113 Mesalazine 500mg/5ml oral suspension

5896141000033114 Sulfasalazine 250mg/5ml oral suspension sugar free

5975741000033118 Sulfadiazine 250mg/5ml oral suspension

6059041000033114 Salofalk 1g suppositories

6059141000033113 Mesalazine 500mg gastro-resistant tablets

6059241000033118 Salofalk 500mg gastro-resistant tablets

6123441000033113 Octasa 800mg MR gastro-resistant tablets

6444841000033112 Pentasa 1g modified-release tablets

6444941000033116 Mesalazine 1g modified-release tablets

6514741000033119 Mesalazine 3g gastro-resistant modified-release granules sachets sugar free

6514841000033112 Salofalk 3g gastro-resistant modified-release granules sachets

8129641000033119 Octasa 400mg MR gastro-resistant tablets

8299241000033114 Sulazine EC 500mg tablets

10042741000033112 Mesalazine 4g modified-release granules sachets sugar free

10042841000033116 Pentasa 4g modified-release granules sachets

12578841000033110 Mesalazine 1g gastro-resistant tablets

12578941000033120 Salofalk 1g gastro-resistant tablets

13179641000033116 Mesalazine 1.6g gastro-resistant tablets

13179741000033112 Octasa 1600mg MR gastro-resistant tablets

13345841000033114 Zintasa 400mg EC tablets

13810741000033120 Octasa 1g suppositories

## Immunomodulators

**Prodcodeid Product name**

98641000033119 Azathioprine 25mg tablets

98741000033111 Azathioprine 50mg tablets

902041000033117 Mercaptopurine 50mg tablets

1403941000033113 Tacrolimus 1mg capsules

1404041000033110 Tacrolimus 5mg capsules

2099141000033117 Tacrolimus 500microgram capsules

2873741000033112 Mercaptopurine 50mg/5ml oral suspension

3050441000033115 Azathioprine 20mg/5ml oral suspension

3086941000033119 Ciclosporin 10mg capsules

3087041000033118 Ciclosporin 25mg capsules

3087141000033119 Ciclosporin 100mg capsules

3087241000033114 Ciclosporin 50mg capsules

3087341000033116 Ciclosporin 100mg/ml oral solution sugar free

3134741000033111 Mercaptopurine 20mg/ml oral suspension

3151941000033113 Azathioprine 50mg/5ml oral suspension

3152241000033110 Tacrolimus 5mg/5ml oral suspension

3188041000033113 Azathioprine 10mg capsules

3216241000033117 Azathioprine 25mg/5ml oral suspension

3216341000033110 Tacrolimus 2.5mg/5ml oral suspension

3282641000033115 Mercaptopurine 10mg capsules

4178541000033110 Tacrolimus 500microgram modified-release capsules

4178641000033111 Tacrolimus 1mg modified-release capsules

4178741000033119 Tacrolimus 5mg modified-release capsules

5131741000033116 Tacrolimus 3mg modified-release capsules

5413541000033116 Tacrolimus 200microgram granules sachets sugar free

5413641000033115 Tacrolimus 1mg granules sachets sugar free

5891341000033119 Azathioprine 50mg/5ml oral solution

5989441000033119 Azathioprine 100mg/5ml oral suspension

5999541000033119 Tacrolimus 2.5mg/5ml oral solution

6066841000033115 Azathioprine 30mg/5ml oral suspension

8493941000033115 Azathioprine 250mg/5ml oral solution

9158841000033114 Mercaptopurine 10mg tablets

9852141000033112 Azathioprine 75mg/5ml oral suspension

10209841000033112 Tacrolimus 750microgram modified-release tablets

10209941000033116 Tacrolimus 1mg modified-release tablets

10210141000033110 Tacrolimus 4mg modified-release tablets

10253441000033116 Tacrolimus 2mg capsules

10253541000033116 Tacrolimus 750microgram capsules

10801841000033116 Mercaptopurine 75mg tablets

11807941000033110 Mercaptopurine 25mg tablets

13317541000033112 Tacrolimus 2mg modified-release capsules

## Advanced therapies

**Prodcodeid Productname**

2994841000033115 Humira 40mg/0.8ml solution for injection pre-filled syringes

4042641000033119 Humira 40mg/0.8ml solution for injection pre-filled pens

6467841000033113 Humira 40mg/0.8ml solution for injection vials

11693141000033110 Humira 40mg/0.4ml solution for injection pre-filled syringes

11693241000033116 Humira 40mg/0.4ml solution for injection pre-filled pens

12512541000033116 Humira 20mg/0.2ml solution for injection pre-filled syringes

12873241000033116 Humira 80mg/0.8ml solution for injection pre-filled pens

12873341000033110 Humira 80mg/0.8ml solution for injection pre-filled syringes

12712841000033116 Adalimumab 20mg/0.4ml solution for injection pre-filled syringes

11693041000033112 Adalimumab 40mg/0.4ml solution for injection pre-filled disposable devices

11692941000033118 Adalimumab 40mg/0.4ml solution for injection pre-filled syringes

2994741000033113 Adalimumab 40mg/0.8ml solution for injection pre-filled syringes

4042541000033115 Adalimumab 40mg/0.8ml solution for injection pre-filled disposable devices

6467741000033115 Adalimumab 40mg/0.8ml solution for injection vials

12873041000033112 Adalimumab 80mg/0.8ml solution for injection pre-filled disposable devices

12873141000033112 Adalimumab 80mg/0.8ml solution for injection pre-filled syringes

12683741000033114 Amgevita 40mg/0.8ml solution for injection pre-filled pens

12685541000033116 Amgevita 40mg/0.8ml solution for injection pre-filled syringes

12873441000033116 Hyrimoz 40mg/0.8ml solution for injection pre-filled pens

12873641000033120 Imraldi 40mg/0.8ml solution for injection pre-filled pens

12873541000033116 Hyrimoz 40mg/0.8ml solution for injection pre-filled syringes

12873741000033112 Imraldi 40mg/0.8ml solution for injection pre-filled syringes

13479441000033116 Idacio 40mg/0.8ml solution for injection vials

13479841000033118 Idacio 40mg/0.8ml solution for injection pre-filled pens

13480141000033112 Idacio 40mg/0.8ml solution for injection pre-filled syringes

5989541000033118 Simponi 50mg/0.5ml solution for injection pre-filled syringes

9069241000033120 Simponi 100mg/1ml solution for injection pre-filled pens

5989641000033117 Simponi 50mg/0.5ml solution for injection pre-filled pens

12378941000033116 Golimumab 100mg/1ml solution for injection pre-filled disposable devices

12379041000033112 Golimumab 50mg/0.5ml solution for injection pre-filled disposable devices

12379141000033112 Golimumab 50mg/0.5ml solution for injection pre-filled syringes

1916141000033115 Infliximab 100mg powder for solution for infusion vials

1916241000033110 Remicade 100mg powder for concentrate for solution for infusion vials

13443941000033112 Remsima 120mg/1ml solution for injection pre-filled syringes

13443841000033116 Remsima 120mg/1ml solution for injection pre-filled pens

13443741000033110 Infliximab 120mg/1ml solution for injection pre-filled syringes

12886741000033110 Zessly 100mg powder for concentrate for solution for infusion vials

13443641000033118 Infliximab 120mg/1ml solution for injection pre-filled disposable devices

10213341000033118 Remsima 100mg powder for concentrate for solution for infusion vials

10229141000033112 Inflectra 100mg powder for concentrate for solution for infusion vials

11749341000033114 Flixabi 100mg powder for concentrate for solution for infusion vials

10644841000033118 Ustekinumab 90mg/1ml solution for injection pre-filled syringes

4937341000033118 Stelara 45mg/0.5ml solution for injection vials

4937241000033111 Ustekinumab 45mg/0.5ml solution for injection vials

5706041000033114 Ustekinumab 45mg/0.5ml solution for injection pre-filled syringes

5706141000033113 Stelara 45mg/0.5ml solution for injection pre-filled syringes

10644941000033114 Stelara 90mg/1ml solution for injection pre-filled syringes

11898541000033118 Ustekinumab 130mg/26ml solution for infusion vials

11898641000033116 Stelara 130mg/26ml concentrate for solution for infusion vials

9301241000033116 Vedolizumab 300mg powder for solution for infusion vials

9301441000033120 Entyvio 300mg powder for concentrate for solution for infusion vials

13510241000033112 Vedolizumab 108mg/0.68ml solution for injection pre-filled syringes

13510341000033120 Vedolizumab 108mg/0.68ml solution for injection pre-filled disposable devices

13510541000033114 Entyvio 108mg/0.68ml solution for injection pre-filled pens

13974041000033112 Tofacitinib 1mg/ml oral solution sugar free

13426941000033116 Xeljanz 11mg modified-release tablets

13426841000033112 Tofacitinib 11mg modified-release tablets

12635441000033112 Tofacitinib 10mg tablets

12635541000033114 Xeljanz 10mg tablets

12205541000033120 Xeljanz 5mg tablets

12205341000033114 Tofacitinib 5mg tablets
